# Californium-252 neutron intracavity brachytherapy alone for T1N0 low-lying rectal adenocarcinoma: A definitive anal sphincter-preserving radiotherapy

**DOI:** 10.1038/srep40619

**Published:** 2017-01-17

**Authors:** Yanli Xiong, Jinlu Shan, Jia Liu, Kewei Zhao, Shu Chen, Wenjing Xu, Qian Zhou, Mei Yang, Xin Lei

**Affiliations:** 1 Cancer Center, Daping Hospital and Research Institute of Surgery, Third Military Medical University, Chongqing, China; 2 Neutron Intracavity Brachytherapy Center, Daping Hospital and Research Institute of Surgery, Third Military Medical University, Chongqing, China

## Abstract

This study evaluated the 4-year results of 32 patients with T1N0 low-lying rectal adenocarcinoma treated solely with californium-252 (Cf-252) neutron intracavity brachytherapy (ICBT). Patients were solicited into the study from January 2008 to June 2011. All the patients had refused surgery or surgery was contraindicated. The patients were treated with Cf-252 neutron ICBT using a novel 3.5-cm diameter off-axis 4-channel intrarectal applicator designed by the authors. The dose reference point was defined on the mucosa surface, with a total dose of 55–62 Gy-eq/4 f (13–16 Gy-eq/f/wk). All the patients completed the radiotherapy in accordance with our protocol. The rectal lesions regressed completely, and the acute rectal toxicity was mild (≤G2). The 4-year local control, overall survival, disease-free survival, and late complication (≥G2) rates were 96.9%, 90.6%, 87.5% and 15.6%, respectively. No severe late complication (≥G3) occurred. The mean follow-up was 56.1 ± 16.0 months. At the end of last follow-up, 29 patients remained alive. The mean survival time was 82.1 ± 2.7 months. Cf-252 neutron ICBT administered as the sole treatment (without surgery) for patients with T1N0 low-lying rectal adenocarcinoma is effective with acceptable late complications. Our study and method offers a definitive anal sphincter-preserving radiotherapy for T1N0 low-lying rectal adenocarcinoma patients.

From 2009 to 2011 in China, colorectal cancer was the second most common malignancy in men and the third most common in women. New colorectal cancer cases and associated deaths were estimated at 376.3 thousand and 191.0 thousand, respectively^1^. The reasons for the rising incidence in colorectal cancer are largely unknown, but nevertheless in China it is recognized as an important health problem.

It is interesting to note that in different parts of the world, the ratio of colon-to-rectal cancer varies. In western countries colon cancer is more common than rectal, but in Asian countries the rates of these 2 diseases are similar^2^. Over the past two decades the successful implementation of screening programs around the world has increased the number of diagnoses of early stage rectal cancer. Even in some districts and provinces of China, screening has led to reduced mortalities^3^,^4^,^5^.

The treatment of rectal cancer varies, depending on its clinical stage and location. Although total mesorectal excision can decrease the local recurrence rate and improve long-term survival, the quality of life for these patients with a stoma is poor^6^. Over the past 2 decades, local excision for T1 stage rectal cancer has been increasingly used to preserve the anal sphincter and diminish postoperative morbidity^7^,^8^,^9^,^10^. As surgical experience has increased, limited excisions have obtained good functional results^11^. However, general anesthesia is mandatory for this procedure, and can be difficult to manage in old or fragile patients. The tumor location and the differentiation status of the tumor cells also influence indications for local excision, and the risk of fistula remains^12^,^13^,^14^,^15^.

Many patients with T1 low rectal adenocarcinoma (<6 cm from the anal verge) are not indicated for local excision, or are not otherwise operable due to generally poor condition (e.g., those with low performance status, severe comorbidity, and the elderly). Yet conventional fractioned radiation is rarely given to these patients, because rectal adenocarcinoma is not sensitive enough.

An alternative treatment is contact radiotherapy, with or without external beam radiotherapy, or a brachytherapy boost via iridium-192 wires, or both^16^,^17^,^18^,^19^. It is well recognized that contact X-ray endocavitary radiation can achieve ideal results for T1N0 rectal adenocarcinoma^20^,^21^, and can spare the sphincter. However, very few large randomized trials have been conducted to confirm definitively the efficacy of contact X-ray for T1-stage rectal lesions^18^.

The discovery of californium-252 (Cf-252), a source of neutron/gamma radiation, has allowed use of neutrons in tumor brachytherapy^22^,^23^. Although Cf-252 neutron intracavity brachytherapy (ICBT) has been reported for rectal cancer, there have been no definitive studies, even for T1 low rectal cancer^24^.

Thus, our present study assessed the 4-year results of 32 patients with T1N0 low rectal adenocarcinoma, who underwent only Cf-252 neutron ICBT using off-axis applicators. These patients had refused surgery, or who were considered inoperable.

## Methods

The Ethics Review Board of Daping Hospital and Research Institute of Surgery, Third Military Medical University, Chongqing, China approved this study (Study number: 201511). All methods were performed in accordance with the ethical standards laid down in 1964 Declaration of Helsinki and its later amendments. All the patients signed informed consent forms before participating in the study.

### Staging and inclusion criteria

From January 2008 to June 2011, 32 patients with T1-N0 low-lying (i.e., <6 cm from the anal verge) rectal adenocarcinoma were considered for this trial (Table 1). Diagnoses had been performed and stages were adjudged via biopsy and endorectal ultrasonography B, in accordance with the standard of the International Union against Cancer Classification (UICC 2007). All the patients were examined by at least one surgeon and underwent a careful digital rectal examination in the knee-chest position with an empty rectal ampulla, and rigid proctoscopy in the same position. The patients were examined by colonoscopy, pelvic magnetic imaging scan, endoscopic ultrasound, liver ultrasonography, and chest X-ray, and full blood count and serum carcinoembryonic antigen were determined. All of the patients were considered free of metastasis.

Among the 32 patients, 6 were inoperable due to general poor condition, and the other 26 adamantly refused abdominal perineal resection (APR) after extensive consultations with surgeons.

Of note, there were no local excision instruments in western China at that time. In patients with low rectal tumor who are operable, radical radiotherapy is first attempted. In cases of radiotherapy failure or serious late complications, salvage APR may recommended after extensive discussion with surgeons and radiation oncologists.

Local recurrence was defined as a recurred tumor in or close to the primary tumor bed in 3 cm after a clinically complete response.

### Cf-252 neutron ICBT

**Figure 1:**
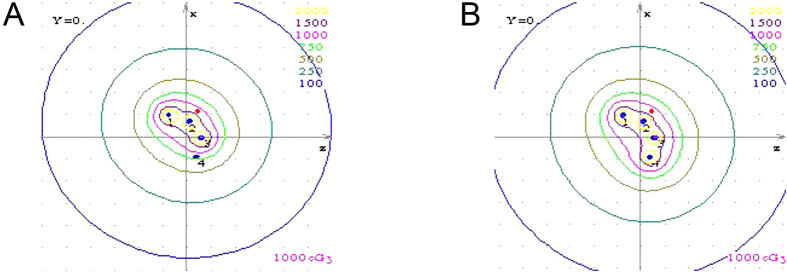
Isodose distribution in transverse section for 252-Cf treatment plan. The reference points were the red points, and was defined on the surface of the mucosa. (**A**) Three channels of the applicator; (**B**) Four channels of the applicator.

Linden LZH-1000 Cf-252 neutron brachytherapy devices (Linden Science and Technology, Shenzhen, China) were used. For neutron ICBT, sealed Cf-252 neutron sources were used. Californium emits both fast neutrons with a mean energy of 2.1–2.3-MeV neutrons, and 0.5–1.0 MeV gamma rays with a half-life of 2.65 years. The specific activity of the neutron sources was 200 μg–520 μg. The patients were administered Cf-252 ICBT as the sole treatment. The reference points were defined on the mucosal surface and the total dosage of ICBT was 55–62 Gy-eq/4 f, 13–16 Gy-eq/f (Fig. 1).

The dose of Cf-252 ICBT was calculated by taking into account the neutron (Dn) and gamma ray (DY) doses. The relative biological effect (RBE) compared to a same dose of gamma radiation was assumed to be 2–6 for neutrons in normal tissues, and was calculated according to the following format:


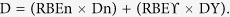


The total dose and dose per fraction were given according to the dose rate (activity) of the Cf-252 neutron sources. Higher activity translated to a higher dose because the relative biological effect was changed along with the activity change.

**Figure 2:**
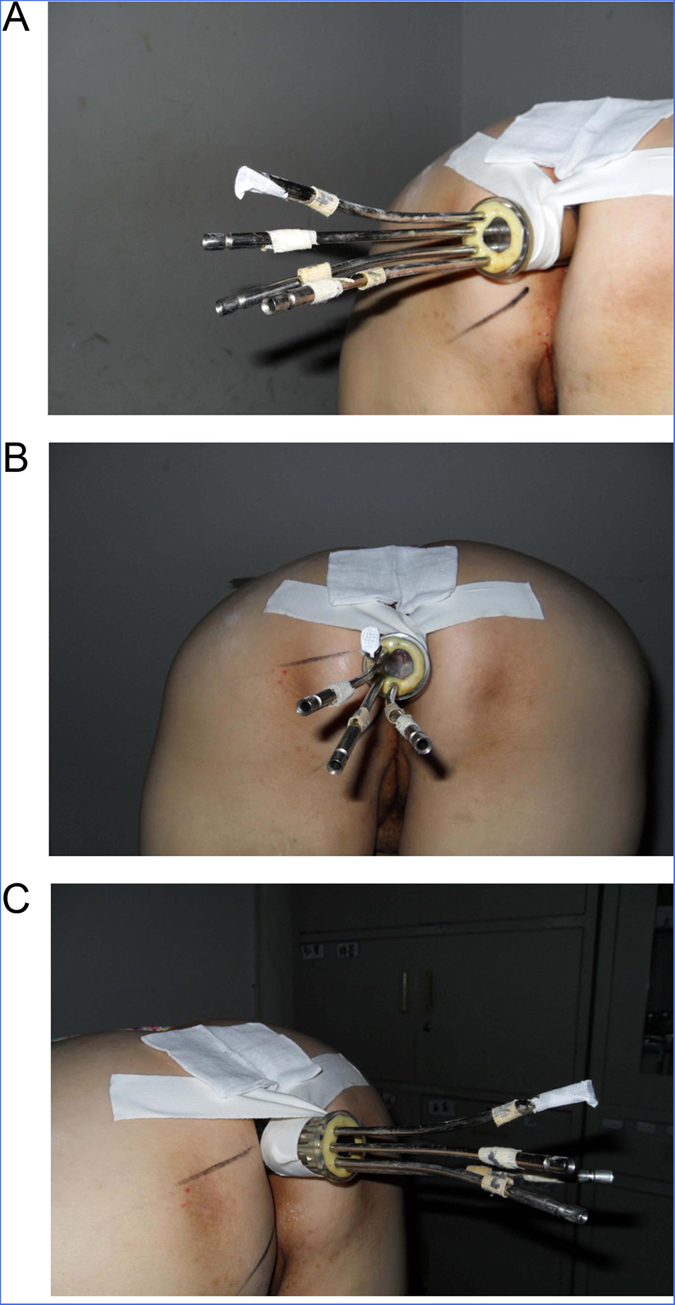
Three or four channels of the off-axis applicator (diameter 3.5 cm) selected based on the dimension of the tumor were inserted to the top of rectum with the patient in the knee-to-chest position. The numbers of dwell points were given according to the length of the tumor and the distance from the anal canal entrance (1 cm beyond the margin of the tumor).

Perianal anesthesia was performed with 20 mL of 2% lidocaine. After the anal sphincter relaxed, a careful digital rectal examination in the knee-chest position with an empty rectal ampulla was performed, and then rigid proctoscopy to determine the range and superior and inferior margins of the tumor from the anal verge. The off-axis applicator with a diameter of 3.5 cm was inserted to the top of the rectum, for preventing the inserted applicator rotating and sliding out of rectum. Three or four channels of the applicator were selected based on the dimension of the tumor. The numbers of dwell points were given according to the length of the tumor and the distance from the anal canal entrance (1 cm beyond the margin of the tumor; Fig. 2).

### Follow-up

The patients were followed for disease status and mortality. The follow-up was performed every 3 months in the first 2 years after completion of treatment and every 6 months thereafter. The patients’ physical and pelvic re-examination was conducted gratis, to prevent loss to follow-up. Each patient’s home, work, and emergency contact addresses were recorded, as well as at least one house telephone number. The patients’ telephone number and address were continually updated, hence no patient was lost to follow-up because of a change of telephone number or address. Outcome events were defined as local or nodal failure and distant metastases.

Early and late toxicity was classified according to the common terminology criteria for adverse events version of the radiation therapy oncology group (RTOG). The anal function was evaluated as excellent, good, fair, or bad using a function questionnaire and the Memorial Sloan-Kettering Cancer Center scoring system.

### Statistical analysis

The SPSS 20.0 software package (SPSS, Chicago, IL) was used to conduct all statistical analyses. Continuous data were summarized as the mean ± standard deviation, and discrete data were counted as frequency. The Kaplan-Meier curve and life tables were used to analyze survival data. Adverse events were evaluated in accordance with the Common Terminology Criteria for Adverse Events of the National Cancer Institute.

## Results

### Characteristics of patients

The mean age of the 32 patients was 60.8 ± 15.3 years (range, 30–86 years; Table 1). Most of the patients were men (62.5%, 20/32). All rectal adenocarcinomas were less than 6 cm from the anal canal entrance, and the ECOG (Eastern Cooperative Oncology Group) score of all these patients was below 2.

### Tumor response

**Figure 3:**
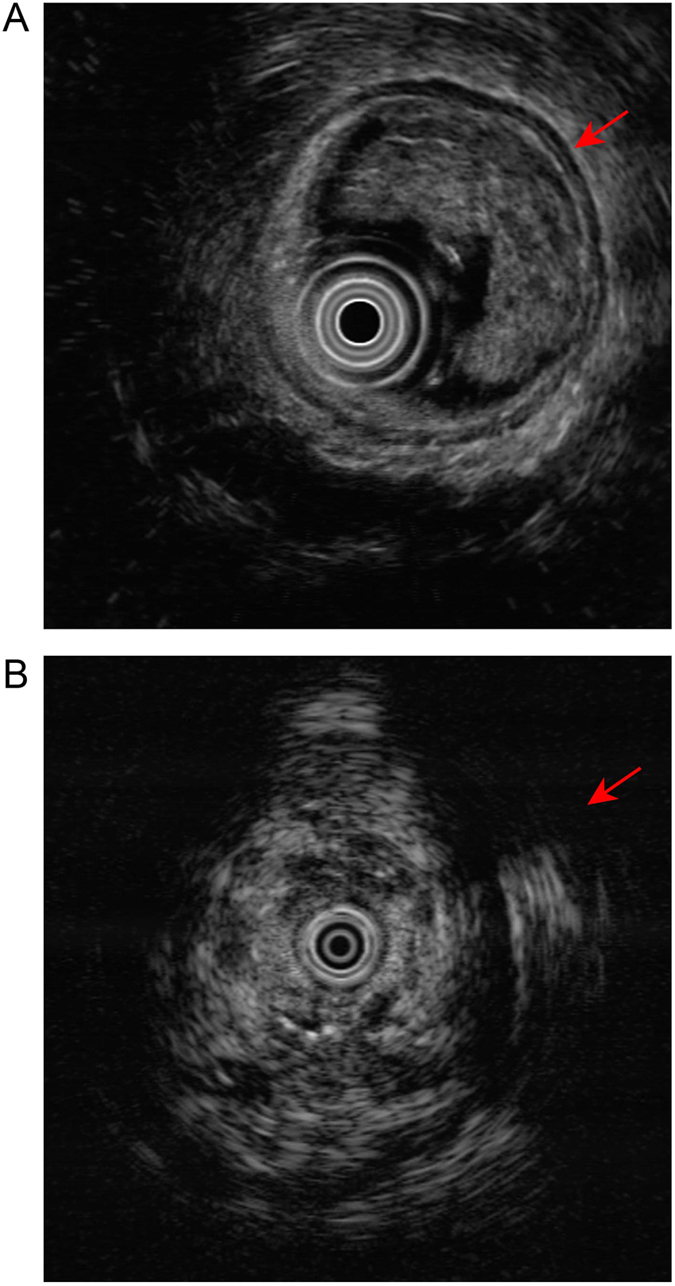
Endoscopic ultrasound images. (**A**) Pre-treatment; (**B**) Six months after radiotherapy.

**Figure 4:**
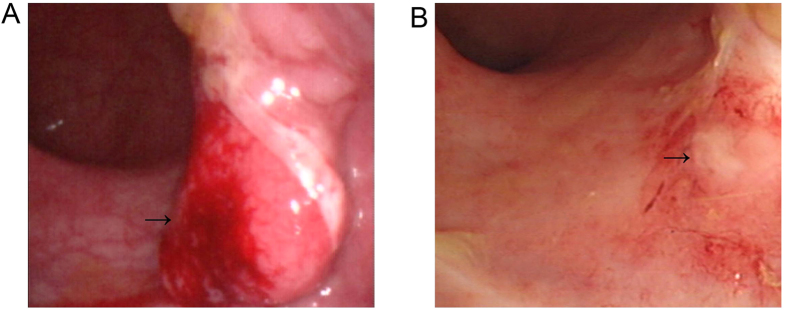
Colonoscopy images. (**A**) Pre-treatment. (**B**) Six months after the combined treatment. There was no residual tumor after treatment.

After ICBT, all patients completed the scheduled treatment and all the rectal lesions regressed completely. The response rate was 100% (32/32). Endoscopic ultrasound and colonoscopy also showed complete regression of the tumor 6 months after treatment (Figs 3 and 4).

### Local control and tumor recurrence

Local recurrence was seen in one patient and the 4-year primary tumor local control rate was 96.9% (31/32). No patient had persistent residual lesion and no patient suffered from lymph node local failure.

### Distant metastases

Distant metastases were detected in 3 patients (1 in the lung and 2 in the liver), who died of distant organ metastasis. In one patient, metastasis was found associated with local failure. One patient suffered from rectal tumor relapse, refused to undergo salvage APR, and died from general deterioration caused by rectal obstruction (Table 2).

### Survival and failure pattern

At the time of the last follow up, 29 patients were still alive with a mean survival time of 82.1 ± 2.7 months. The deaths of the deceased patients were all related to rectal adenocarcinoma. With a mean follow-up time of 56.1 ± 16.0 months, the 4-year overall survival rate was 90.6%, and the disease-free survival rate was 87.5%.

All the patients completed the treatment in accordance with our protocol. The acute side effects of the rectum were mild (G1 and G2).

The rate of late complications (≥G2) was 15.6% for the patients. No patients experienced G3 complications requiring colostomy. Late complications included rectal bleeding resulting from radiation-induced rectal mucosal telangiectasia (1 patient), rectal frequent urgency (tenesmus) due to radiation-induced rectal mucosal ulcer, necrosis (3 patients), and mixed rectal bleeding and tenesmus caused by mucosal telangiectasia plus necrosis (1 patient) (Table 3).

Anal sphincter function was scored as excellent in 26 patients, good in 4 patients, and fair in 2 patients, mainly based on tenesmus.

## Discussion

Radical resection, including total mesorectal excision, is considered the standard surgical treatment for early low-lying rectal cancer. However, a delicate balance exists between quality of life and survival in the treatment of rectal cancer, and the quality of life of patients with a stoma is poor. The accepted alternative treatment is local excision.

Ptok *et al*.^25^ reported that the 5-year local tumor recurrence rate associated with radical resection (6.0%) exceeded that of local excision (2.0%; *P* = 0.049), but tumor-free survival did not differ. De Graaf *et al*.^26^ from the Netherlands found that only local recurrence rates differed between these procedures. Furthermore, most studies show no difference in survival, while compared with radical resection, local excision is associated with lower rates of postoperative complications, postoperative mortality, and dysfunction^27^. You *et al*.^7^ reported the results of a 1989–2003 survey from the National Cancer Database in the United States on the use of local excision. The 5-year survival rate was 81.7% for patients given radical resection and 77.4% for those given local excision, and the survival curves did not statistically differ. Heintz *et al*.^28^ from Germany found no difference in 5-year overall survival rates. These results indicate that the advantages of local excision over radical resection include less damage and more rapid recovery. Thus, local excision can have an important role in the treatment of early-stage rectal cancer.

Indeed, local excision can promote the quality of life of patients with low-lying rectal cancer. However, these surgical procedures require patients to be under anesthesia for at least 1–2 hours, and general anesthesia is not indicated for some patients with poor physical condition, especially the elderly. Alternatively, contact radiotherapy needs only local anesthesia and can be effective for rectal cancer patients at stage T1N0, preserves the anal sphincter, and diminishes postoperative morbidity^18^,^21^,^29^. Several studies confirmed that contact radiotherapy was not inferior to local excision in local control and survival rates^20^,^21^,^23^,^29^,^30^. Yet, large prospective randomized parallel trails are still lacking to confirm efficacy. This is because only a few institutions possess Philips radiographic equipment, as the company has discontinued production.

On the other hand, Cf-252, discovered in 1950, emits a mixed flux of 2.1–2.3 MeV neutrons and 0.5–1.0 MeV high-energy gamma rays. Neutrons are high linear energy transfer radiation with benefits^31^ that include high relative biological effect, low oxygen enhancement ratio, and a high proportion of DNA double-strand breaks in tumor cells^32^,^33^,^34^. It has been found effective as radiotherapy and advantageous for the treatment of radio-resistant advanced or bulky uterine cervical tumors^35^ and T2 low-lying rectal cancer^24^.

While Cf-252 neutron radiation can be beneficial in T1 low-lying rectal cancer, the protection of normal rectal tissue remains a challenge in Cf-252 neutron ICBT. As we know, the tolerated radiation dose of the rectum is inversely proportional to the radiation area (volume), and increasing the dose of radiation is a major strategy making it more effective^36^. That is to say, higher doses can be delivered to the rectum on a smaller volume. Furthermore, a dose of 70–75 Gy was confirmed to be given to a small surface or volume (<100 mm^3^) of the rectum without any severe late effects other than tenesmus and bleeding for prostate cancer. The experience of contact X-ray radiotherapy for T1 rectal cancer also highlights the importance of the dose-volume ratio in late tolerance of the rectum^20^,^37^. A dose of 100–120 Gy can be given to a smaller surface or volume (<30 mm^3^) of the rectum with very few severe late effects. The above arguments suggest that the only way to treat rectal adenocarcinoma with radiotherapy as the sole treatment is to deliver a very high dose to a small volume.

Because the T1N0 rectal lesion, by definition, has only invaded the mucosa and submucosa, neutron ICBT or contact X-ray could be used as the efficient treatment to treat these patients. According to these principles, we designed and manufactured 3.5 cm-diameter off-axis applicators which was awarded patent in China (Patent number: ZL 2006 I 0095108.9) to treat T1N0 rectal tumor. During Cf-252 ICBT, the dose to normal tissue surrounding and contralateral to the lesions is decreased greatly. Furthermore, large-area normal rectal mucosa is spared from a high irradiation dose when the local tumor is controlled by Cf-252 neutrons. Hence, a high local control rate can be achieved with very low rates of serious late complications.

It has been reported by many authors that contact X-ray alone can achieve local control in 81.2–95.5% of T1N0 rectal cancer patients, tolerability is good in most patients, anorectal function is preserved, and the risk of severe late toxic effects is very low (Table 4). Our present results also showed that the 4-year local control and late complications (≥G2) rates for these patients were 96.9% and 15.6%, respectively. Only one patient suffered from relapse of local lesions, and 3 patients died from distant metastases (2 patients in liver, 1 patient in lung). The overall survival and disease-free survival rates were 90.6% and 87.5% respectively, which are not inferior to the results for contact X-ray of T1 stage rectal cancer^20^,^21^,^23^,^29^,^30^. Because Cf-252 neutrons deliver higher penetration relative to 50 kV contact X-ray, it may be a promising option for T1N0 rectal cancer patients who are not fit for surgery.

Although we used 3.5 cm-diameter off-axis applicators, normal rectal mucosa was still exposed to some extent. Hence, the late complication rate in T1 patients (≥G2) was 15.6% after ICBT. However, no severe late complications requiring colostomy (≥G3) occurred, which is not higher than that for T1 patients given contact X-ray only (Table 4). Furthermore, since January 2013 we have injected amifostin into the submucosa surrounding the rectal lesion during every ICBT, and the late complication rate associated with ICBT (given solely) for T1 patients is now lower, and no patients have suffered from late complications (≥G2).

## Conclusion

For patients with T1N0 low-lying rectal adenocarcinoma not given surgery, Cf-252 neutron ICBT with our novel off-axis applicator is effective and has an acceptable rate of late complications. It may be a promising option for these patients. However, a large randomized trial is still needed to compare the outcomes of Cf-252 neutron ICBT to that of total mesorectal excision, local excision, and contact radiotherapy.

## Additional Information

**How to cite this article**: Xiong, Y. *et al*. Californium-252 neutron intracavity brachytherapy alone for T1N0 low-lying rectal adenocarcinoma: A definitive anal sphincter-preserving radiotherapy. *Sci. Rep.* **7**, 40619; doi: 10.1038/srep40619 (2017).

**Publisher's note:** Springer Nature remains neutral with regard to jurisdictional claims in published maps and institutional affiliations.

## Figures and Tables

**Table 1:** Demographic and baseline characteristics of the patients.

		n
Age, y	30–40	4
	41–50	5
	51–60	6
	61–70	7
	71–86	10
Mean age, y	60.8	—
Male/female	—	20/12
Reason for no surgery	Inoperable*	6
	Patient refusal	26
Site	Anterior	6
	Lateral	21
	Posterior	5
Distance from anal canal entrance, cm	1.0–3.0	9
	3.0–4.5	14
	4.5–6.0	9
Mean distance from anal verge, cm	3.7	—
Tumor thickness, cm	0.0–1.0	7
	1.0–2.0	23
	2.0–3.0	2

^*^Due to generally poor physical condition, due to age, performance status, or severe comorbidity.

**Table 2:** Patterns of tumor recurrence in the study participants.

		n (%)
No		8 (87.5)
Yes		4 (12.5)
	Local	1 (3.1)
	Lung	1 (3.1)
	Liver	2 (6.3)
Total		32 (100)

**Table 3:** Late complications (≥G2).

	n (%)
Tenesmus	3 (9.4)
Bleeding	1 (3.1)
Tenesmus + bleeding	1 (3.1)
Total	5 (15.6)

**Table 4:** Results of endocavitary radiation therapy only in the treatment of T1 stage low rectal cancer.

First author, year, ^Ref^	Basic treatment	Patients, n	Local recurrence%	Survival%^a^	Complications n (%)^b^	
De Graaf^26^	Local excision	80	24.0	75.0	NA	
	Radical resection	75	0	71.0	NA	
You^7^	Local excision	561	8.2	77.4	NA	
	Radical resection	465	4.3	81.7	NA	
Ptok^25^	Local excision	105	6.0	83.6	NA	
	Radical resection	312	2.0	91.5	NA	
Heintz^28^	Local excision	46	NA	75.6	NA	
	Radical resection	34	NA	78.1	NA	
Coatmeur^21^	Contact X-ray	80	18.8	76.3	27/124 (21.8)	
Rauch^30^	Contact X-ray	69	15.9	79.7	NA	
Gérard^20^	Contact X-ray	65	6.6	86.8	0/65 (0)	
Schild^23^	Contact X-ray	20	15.0	76.0	5/25 (20.0)	
Papillon^29^ ^c^	Contact X-ray	310	4.5	73.8	NA	
Our present study^d^	252-Cf neutron	32	3.1	90.6	5/30 (15.6)	

^a^Five-year survival.

^b^>G2, i.e., ulceration, rectorrhagia.

^c^This study contained both T1 and T2 patients.

^d^Four-year study.

NA: not available.
